# Associations of low sex hormone‐binding globulin and androgen excess in early pregnancy with fasting and post‐prandial hyperglycaemia, gestational diabetes, and its severity

**DOI:** 10.1002/dmrr.3599

**Published:** 2022-12-19

**Authors:** Sanna Mustaniemi, Laure Morin‐Papunen, Elina Keikkala, Hanna Öhman, Heljä‐Marja Surcel, Risto Kaaja, Mika Gissler, Johan G. Eriksson, Hannele Laivuori, Eero Kajantie, Marja Vääräsmäki

**Affiliations:** ^1^ Department of Obstetrics and Gynaecology PEDEGO Research Unit Medical Research Center Oulu University Hospital University of Oulu Oulu Finland; ^2^ Department of Public Health and Welfare Population Health Unit Finnish Institute for Health and Welfare Oulu Helsinki Finland; ^3^ Biobank Borealis of Northern Finland Oulu University Hospital Oulu Finland; ^4^ Faculty of Medicine Medical Research Center University of Oulu Oulu Finland; ^5^ Institute of Clinical Medicine Internal Medicine Turku University Hospital University of Turku Turku Finland; ^6^ Department of Knowledge Brokers Finnish Institute for Health and Welfare Helsinki Finland; ^7^ Academic Primary Health Care Centre Region Stockholm and Department of Molecular Medicine and Surgery Karolinska Institute Stockholm Sweden; ^8^ Department of General Practice and Primary Health Care Helsinki University Hospital University of Helsinki Helsinki Finland; ^9^ Folkhälsan Research Center Helsinki Finland; ^10^ Department of Obstetrics and Gynecology Human Potential Translational Research Programme Yong Loo Lin School of Medicine National University of Singapore Singapore Singapore; ^11^ Department of Obstetrics and Gynecology Tampere University Hospital and Faculty of Medicine and Health Technology Center for Child, Adolescence and Maternal Health Tampere University Tampere Finland; ^12^ Medical and Clinical Genetics Helsinki University Hospital University of Helsinki Helsinki Finland; ^13^ Institute for Molecular Medicine Finland Helsinki Institute of Life Science University of Helsinki Helsinki Finland; ^14^ Children's Hospital Helsinki University Hospital University of Helsinki Helsinki Finland; ^15^ Department of Clinical and Molecular Medicine Norwegian University of Science and Technology Trondheim Norway

**Keywords:** androgen excess, free androgen index, gestational diabetes, pre‐pregnancy BMI, sex hormone‐binding globulin, testosterone

## Abstract

**Aims:**

We studied whether androgen excess and low sex hormone‐binding globulin (SHBG) measured in early pregnancy are independently associated with fasting and post‐prandial hyperglycaemia, gestational diabetes (GDM), and its severity.

**Materials and Methods:**

This nationwide case–control study included 1045 women with GDM and 963 non‐diabetic pregnant controls. We measured testosterone (T) and SHBG from biobanked serum samples (mean 10.7 gestational weeks) and calculated the free androgen index (FAI). We first studied their associations with GDM and secondly with the type of hyperglycaemia (fasting, 1 and 2 h glucose concentrations during the oral glucose tolerance test), early‐onset GDM (<20 gestational weeks) and the need for anti‐diabetic medication.

**Results:**

After adjustments for gestational weeks at sampling, pre‐pregnancy BMI, and age, women with GDM had 3.7% (95% CI 0.1%–7.3%) lower SHBG levels, 3.1% (95% CI 0.1%–6.2%) higher T levels, and 4.6% (95% CI 1.9%–7.3%) higher FAI levels than controls. SHBG was inversely associated with fasting glucose, whereas higher FAI and T were associated with higher post‐prandial glucose concentrations. Women with early‐onset GDM had 6.7% (95% CI 0.7%–12.7%) lower SHBG levels and women who needed insulin for fasting hyperglycaemia 8.7% (95% CI 1.8%–14.8%) lower SHBG levels than other women with GDM.

**Conclusions:**

Lower SHBG levels were associated especially with early‐onset GDM, higher fasting glucose and insulin treatment, whereas androgen excess was associated with higher post‐prandial glucose values. Thus, a low SHBG level may reflect the degree of existing insulin resistance, while androgen excess might impair post‐prandial insulin secretion.

## 1 INTRODUCTION

Gestational diabetes (GDM) is becoming more common, affecting 10%–25% of pregnancies depending on study populations, screening strategies, and diagnostic criteria applied.^1^, ^2^ It is strongly related to insulin resistance and adiposity; therefore, it is often the first signal of an increased risk of subsequent diabetes, metabolic syndrome, and other cardiovascular risks.^3^, ^4^, ^5^, ^6^, ^7^ Gradually increasing insulin resistance is physiological during a normal pregnancy.^3^, ^5^ Amongst women with GDM, insulin resistance is common before pregnancy, and when pancreatic beta cells are incapable of compensating for the increased need for insulin, hyperglycaemia results.^3^, ^5^ Particularly, women with fasting hyperglycaemia or early‐onset GDM are more insulin resistant, and they often need anti‐diabetic medication.^8^, ^9^, ^10^ These women are at the greatest risk for type 2 diabetes in the long term.^6^, ^10^

The underlying early mechanisms leading to metabolic disturbances and abnormalities in glucose metabolism are complex and not fully understood. In women, low sex hormone‐binding globulin (SHBG) levels are associated with insulin resistance, compensatory hyperinsulinaemia, hyperglycaemia, adiposity, and androgen excess, which are most commonly related to polycystic ovary syndrome (PCOS).^11^, ^12^, ^13^ Insulin resistance and adiposity stimulate insulin secretion, which, in turn, activates ovarian androgen production.^12^ Studies indicate that hyperinsulinaemia, androgen excess and glucose‐induced lipogenesis inhibit hepatic SHBG synthesis.^12^, ^14^, ^15^ This leads to lower circulating SHBG concentrations in which the binding capacity of testosterone (T) is decreased and the amount of biologically active T is increased.^12^ Androgen excess (characterised by both elevated serum total T concentrations and an increased T‐to‐SHBG ratio, defined as the free androgen index [FAI]) further aggravates insulin resistance and compensatory hyperinsulinaemia, leading to a vicious cycle. Rodent models also suggest that, independent of insulin resistance, androgen excess causes chronic androgen receptor activation in beta cells, promoting insulin hypersecretion, and secondary beta cell failure.^16^

Pregestational or first‐trimester low SHBG levels have been associated with subsequent GDM in many^17^, ^18^, ^19^, ^20^, ^21^, ^22^, ^23^ but not in all studies.^22^, ^24^, ^25^, ^26^ It has also been associated with the need for insulin therapy amongst women with GDM.^27^ Nevertheless, most of the previous studies performed in pregnant women had small sample sizes and did not adjust for pre‐pregnancy BMI, which is strongly associated with low SHBG and GDM.^18^, ^21^, ^23^, ^27^ Furthermore, the role of early pregnancy androgen excess in subsequent GDM has been little studied. In two studies, first‐trimester T was found to be slightly higher in women who subsequently developed GDM, although the results were not adjusted for pre‐pregnancy BMI.^17^, ^28^

Therefore, we first aimed to study whether lower SHBG levels and higher T and FAI levels in early pregnancy are associated with GDM, and secondly with the type of hyperglycaemia (fasting and post‐prandial), and the severity of GDM defined by early‐onset disease (<20 weeks of gestation) or the need for anti‐diabetic medication. We clarified whether these associations could be explained by potential confounders, such as pre‐pregnancy BMI.

## 2 MATERIALS AND METHODS

### 2.1 Study population

This case–control study is based on the clinical genetic arm of the Finnish Gestational Diabetes study (FinnGeDi), which includes 1146 women with GDM and 1066 non‐diabetic controls and their newborns^29^, ^30^ (Figure S1). The participants were recruited between 1 February 2009 and 31 December 2012 in seven Finnish delivery hospitals, each serving a specific geographical catchment area. Women with pregestational diabetes and non‐singleton pregnancies were excluded. The participants with GDM were recruited at the delivery units as they entered to give birth. The next consenting non‐diabetic mother giving birth in the same unit was invited as a control. Signed informed consent was obtained from all the participants. The Ethics Committee of Northern Ostrobothnia Hospital District approved the study.

### 2.2 Clinical data

Extensive data from hospital and maternal welfare clinic records were collected.^29^, ^30^ The participants completed background questionnaires, which included information on their lifestyle factors and family and medical histories. In total, 1030 women (89.9%) from the GDM group and 935 women (87.7%) from the control group returned the questionnaire. Register data were obtained from the Finnish Medical Birth Register (FMBR), which contained data on pregnancy, delivery and perinatal health.

A comprehensive screening policy for GDM based on the Finnish National Current Care Guidelines was used, according to which a 2 h 75 g oral glucose tolerance test (OGTT) was recommended for all pregnant women at 24–28 weeks of gestation, except those with a very low risk for GDM, that is, normal‐weight primiparous women under 25 years without a family history of diabetes and normal‐weight multiparous women under 40 years without a history of GDM or macrosomic births.^31^ For high‐risk women (history of GDM, BMI ≥35 kg/m^2^, glucosuria, family history of type 2 diabetes, or PCOS), the OGTT was recommended at 12–16 weeks of gestation and repeated at 24–28 weeks of gestation if the first OGTT was normal. The diagnosis of GDM was set if a woman had one or more abnormal values in the OGTT. The cut‐off values for venous plasma glucose were ≥5.3 mmol/L after fasting, ≥10.0 mmol/L at 1 h and ≥8.6 mmol/L at 2 h after the glucose load. The participants' GDM status was confirmed from their medical records. Due to the screening strategy,^31^ OGTT was not performed for 37.2% (*n* = 358) of controls.

Information on maternal age at delivery, parity, and smoking during pregnancy was obtained from the FMBR. Self‐reported maternal height, pre‐pregnancy weight, and weight measured at the first and the last antenatal visit were obtained from the maternity welfare clinic records. BMI was calculated (kg/m^2^) using height and pre‐pregnancy weight. The educational attainment of the participants was obtained from a questionnaire and categorised as basic or less, upper secondary, lower‐level tertiary, or upper‐level tertiary. Gestational weight gain was calculated as the difference between the pre‐pregnancy weight and the weight at the last antenatal visit (≥35 weeks of gestation).

The definition of PCOS was based on self‐reported prior diagnosis (*n* = 124) and/or PCOS symptoms (*n* = 87) in line with the Rotterdam criteria.^30^, ^32^ Women with PCOS symptoms (*n* = 174) were characterised by typical symptoms of both oligomenorrhoea (menstrual cycle more than 35 days at least twice a year without hormonal contraceptives) and hirsutism (excessive body hair and/or removing facial hair at least four times a month).

Hypertensive pregnancy complications included chronic hypertension, pre‐eclampsia, and gestational hypertension. Chronic hypertension was defined as systolic blood pressure ≥140 mmHg and/or diastolic blood pressure ≥90 mmHg detected repeatedly before 20 weeks of gestation or if the participant used antihypertensive medication before 20 weeks of gestation. Pre‐eclampsia was defined as systolic blood pressure ≥140 mmHg and/or diastolic blood pressure ≥90 mmHg measured after 20 weeks of gestation with proteinuria (≥0.3 g protein/24 h urine specimen or two ≥1+ readings on a dipstick test). Gestational hypertension was considered when hypertension appeared after 20 weeks of gestation without proteinuria.

Data on the birth weight, birth length, head circumference, and sex of the newborn were obtained from the FMBR. The birth weight standard deviation (SD) score is a sex‐ and parity‐specific parameter estimating birth weight and length in singletons born at 23–43 gestational weeks, according to Finnish standards.^33^ Large for gestational age (LGA) was defined as birth weight >+2 SD for sex and gestational age.

### 2.3 Serum samples and laboratory analysis

Data on maternal early pregnancy circulating total T and SHBG concentrations were collected using serum samples from the Finnish Maternity Cohort, which is a nationwide biobank comprising leftover serum samples from routine infectious disease screening during early pregnancy (10–12 weeks of gestation). Therefore, the timing of the blood drawn was not standardised and fasting before sampling was not required. The samples were stored at −25°C in the Biobank Borealis of Northern Finland.

Samples were available from 2030 (91.8%) participants. Those participants with a missing sample (total *n* = 182, *n* = 124 no sample in the biobank, *n* = 58 sample has run out) and those with samples drawn ≥20 + 0 weeks of gestation (*n* = 22) were excluded, rendering a total sample size of 2008 (Figure S1). The mean gestational age at sampling was 10.7 (SD 2.1) weeks of gestation.

The samples were analysed for serum total T concentrations (nmol/L) (ARCHITECT 2nd Generation Testosterone Assay) and SHBG concentrations (nmol/L) (ARCHITECT SHBG Assay) using a chemiluminescence microparticle immunoassay on the Architect i2000SR automatic immunoassay analyser (Abbott Diagnostics). The 2nd Generation Testosterone Assay method is capable of detecting low T concentrations, which are typical for women, and is comparable to liquid chromatography‐tandem mass spectrometry.^34^, ^35^ Internal control samples of pooled serum derived from pregnant women in the first and third trimesters were included in each set of daily assays. The coefficient of variation (CV, SD/mean) was derived from repeated internal control samples. The CV for T was 4.8% (range 2.6–3.1 nmol/L) for the first trimester and 3.5% (range 3.1–3.5 nmol/L) for the third trimester. The CV for SHBG was 5.5% (range 140.3–164.5 nmol/L) for the first trimester and 6.5% (range 473.0–560.9 nmol/L) for the third trimester. T and SHBG concentrations were used to calculate the FAI (T nmol/L × 100/SHBG nmol/L). Early pregnancy T and FAI levels were used as markers of androgen excess.

Of the 2212 participants, a total of 2008 (90.8%) women with early pregnancy laboratory data were included. Hence, 1045 women with GDM and 963 non‐diabetic controls were included in the analysis (Figure S1). When the associations of SHBG, T, and FAI were estimated with the type of hyperglycaemia and the severity of GDM, only women with GDM were included in the analysis.

### 2.4 Statistical methods

The characteristics of the participants in the GDM and non‐diabetic groups were compared using the Student's *t*‐test for continuous variables (expressed as means and SDs) or the χ^2^ test for categorical variables (expressed as frequencies), as well as using logistic regression. All laboratory data were evaluated as categorical and continuous variables. The levels of T, SHBG, and FAI were logarithmically transformed to attain normality, and the distributions of these variables were described in terms of geometric means and geometric standard deviations. We used linear regression models (mean differences with 95% CIs) to compare the laboratory parameters as continuous variables between the women with GDM and the controls. To facilitate comparison, we used SHBG, T, or FAI as the dependent variable. The levels of T and SHBG were stratified by tertiles. The highest tertile of SHBG and the lowest tertile of T or the FAI were used as references. First, logistic regressions (ORs with 95% CIs) were calculated to estimate the associations of the SHBG, T, and FAI tertiles with GDM. Secondly, when the associations of SHBG, T, and FAI with the type of hyperglycaemia and the severity of GDM were assessed, only women with GDM were included in the linear regression analysis. Again, SHBG, T, or FAI was used as the dependent variable.

Categorical adjusting variables were entered as dummy variables, with a separate dummy for missing values. Model 1 was unadjusted. Model 2 included adjustments for gestational weeks at the sampling, pre‐pregnancy BMI, and maternal age at delivery. In Model 3, adjustments included the variables in Model 2 and smoking during pregnancy, educational attainment, parity, delivery hospital, and sex of the newborn. The final Model 4 included the variables in Model 3, hypertensive pregnancy complications and gestational weight gain. Additional analyses were conducted after excluding those participants with PCOS and after excluding eight women with *T* > 10.0 nmol/L. All *p* values were two sided.

The study was powered according to differences in SHBG, T, or FAI as continuous outcome variables. With a power of 0.80, a significance level of 0.05, we were able to detect a 0.13 SD difference between women with GDM and the controls.

## 3 RESULTS

### 3.1 Characteristics of the study population

The women with GDM were older, less often primiparous, and had a higher pre‐pregnancy BMI but lower gestational weight gain than the non‐diabetic controls. The prevalence of PCOS was higher amongst women with GDM, but the difference was related to their higher age and pre‐pregnancy BMI. The women with GDM had more hypertensive pregnancy complications than the controls, and the incidence of chronic hypertension and pre‐eclampsia was higher (Table 1).

**TABLE 1:** Maternal and perinatal characteristics of the participants (*n* = 2008)

	GDM *n* = 1045		Control *n* = 963			
Characteristic	Mean (SD)/*n* (%)	No. of missing	Mean (SD)/*n* (%)	No. of missing	*p* value^a^	*p* value^b^
Age at delivery, years	32.1 (5.4)	0	29.5 (5.2)	0	<0.001	
Parity, *n*	1.3 (2.0)	0	1.1 (1.8)	0	0.040	
Primiparous, *n* (%)	447 (42.8%)	0	598 (62.1%)	0	0.025	
Pre‐pregnancy weight, kg	76.5 (17.1)	1	64.6 (12.2)	0	<0.001	<0.001^c^
Height, cm	164.9 (5.9)	0	165.4 (5.8)	0	0.030	
Pre‐pregnancy BMI, kg/m^2^	28.1 (6.0)	1	23.6 (4.1)	0	<0.001	<0.001^c^
Gestational weight gain, kg	12.3 (5.8)	83	14.8 (5.0)	30	<0.001	<0.001^c^
Educational attainment		104		117	0.015	
Basic or less, *n* (%)	64 (6.8%)	0	36 (4.3%)	0		
Upper secondary, *n* (%)	441 (46.9%)	0	388 (45.9%)	0		
Lower‐level tertiary, *n* (%)	246 (26.1%)	0	209 (24.7%)	0		
Upper‐level tertiary, *n* (%)	190 (20.2%)	0	213 (25.2%)	0		
Smoking during pregnancy, *n* (%)	169 (16.2%)	3	143 (14.9%)	1	0.404	
PCOS, *n* (%)	97 (10.5%)	117	63 (7.5%)	124	0.032	0.927^d^
Chronic hypertension, *n* (%)	168 (16.1%)	1	44 (4.6%)	0	<0.001	0.001^d^
Gestational hypertension, *n* (%)	216 (20.7%)	1	140 (14.5%)	0	<0.001	0.286^d^
Pre‐eclampsia, *n* (%)	61 (5.8%)	1	24 (2.5%)	0	<0.001	0.029^d^
Induction of labour, *n* (%)	473 (45.3%)	0	309 (32.1%)	0	<0.001	0.008^d^
Caesarean section, *n* (%)	210 (20.1%)	0	132 (13.7%)	0	<0.001	
Gestational age at delivery, weeks	39.6 (1.4)	0	40.1 (1.4)	0	<0.001	<0.001^e^
<37 + 0 weeks, *n* (%)	39 (3.7%)	0	20 (2.1%)	0	0.028	0.218^e^
Birth weight, g	3646 (505)	0	3575 (500)	0	0.002	<0.001^f^
Birth weight SD score	0.3 (1.1)	0	−0.1 (1.0)	0	<0.001	<0.001^f^
LGA, >+2 SD, *n* (%)	60 (5.7%)	0	22 (2.3%)	0	<0.001	0.108^d^
Early‐onset GDM (<20 weeks of gestation), *n* (%)	300 (30.3%)	55	−(0.0%)	0		
Use of any anti‐diabetic medication (insulin and/or metformin), *n* (%)	197 (19.2%)	21	−(0.0%)	0		
Insulin, *n* (%)	184 (18.0%)	21				
Short‐acting, *n* (%)	28 (15.2%)					
Long‐acting, *n* (%)	106 (57.6%)					
Both, *n* (%)	47 (25.5%)					
Not specified, *n* (%)	3 (1.6%)					
Metformin, *n* (%)	22 (2.2%)	28				

Abbreviations: GDM, gestational diabetes; LGA, large for gestational age; PCOS, polycystic ovary syndrome.

^a^ Unadjusted *p* values based on the Student's *t*‐test or the χ^2^ test.

^b^ Adjusted *p* values by logistic regression.

^c^ Adjusted for parity and maternal age.

^d^ Adjusted for parity, maternal age and pre‐pregnancy BMI.

^e^ Adjusted for parity, maternal age, pre‐pregnancy BMI, induction of labour (yes/no) and hypertensive pregnancy complications.

^f^ Adjusted for parity, maternal age, gestational age at delivery, pre‐pregnancy BMI and hypertensive pregnancy complications.

### 3.2 Associations of SHBG, T, and FAI with GDM

**FIGURE 1:**
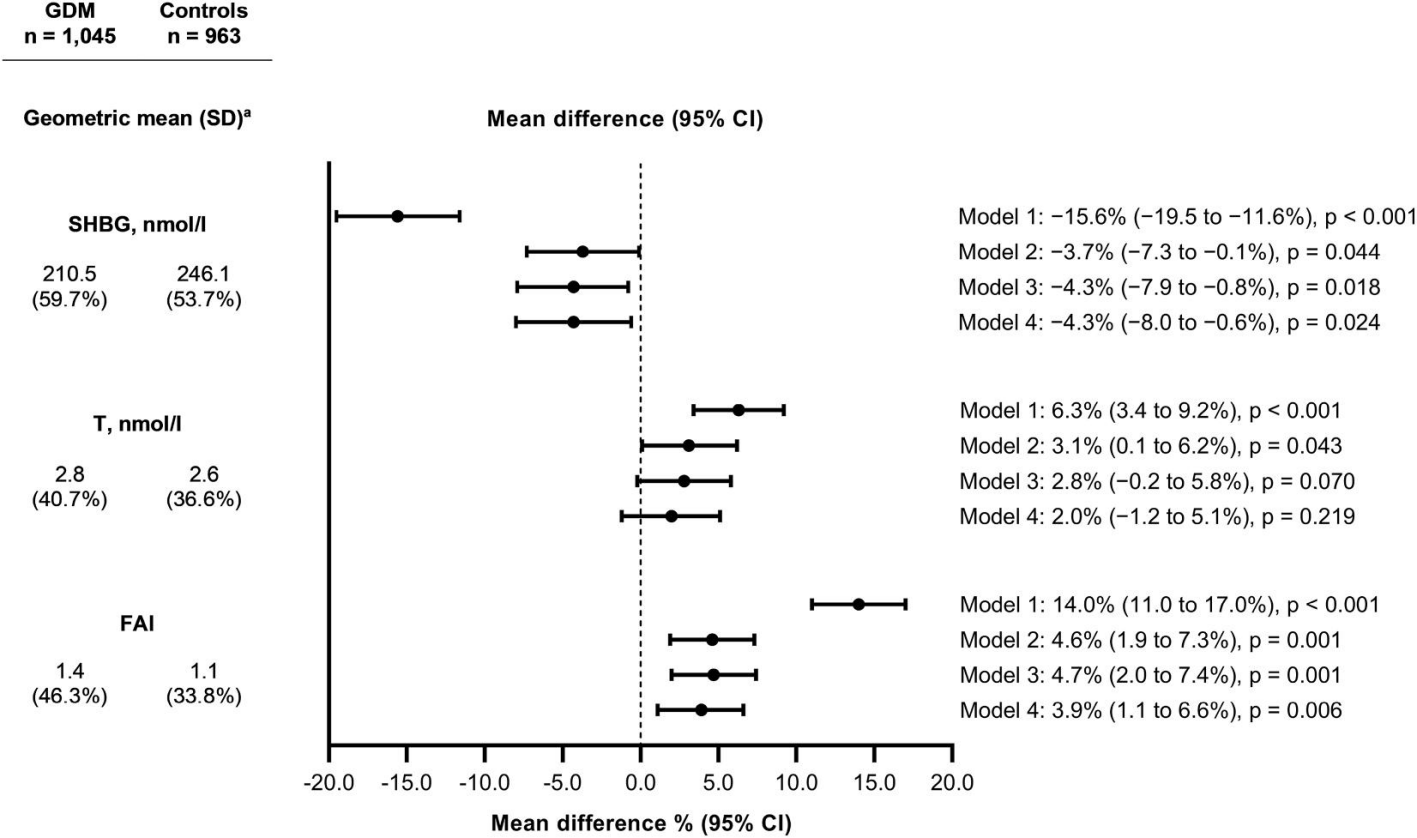
Geometric mean values^a^ of sex hormone‐binding globulin (SHBG), testosterone (T) and free androgen index (FAI) and the mean differences between the women with gestational diabetes (GDM) and the non‐diabetic controls (*n* = 2008). ^a^The geometric mean is the *n*th root of the product of *n* values. Geometric SDs correspond to the percent increase in the variable corresponding to a change of one SD unit in the logarithm of the variable. Model 1 was unadjusted. Model 2 was adjusted for gestational weeks at the sampling, pre‐pregnancy BMI, and maternal age. Model 3 was adjusted for the variables in Model 2 and for smoking during pregnancy, educational attainment, parity, delivery hospital, and sex of the newborn. Model 4 was adjusted for the variables in Model 3 and for hypertensive pregnancy complications and gestational weight gain. FAI, free androgen index; GDM, gestational diabetes; SHBG, sex hormone‐binding globulin

**FIGURE 2:**
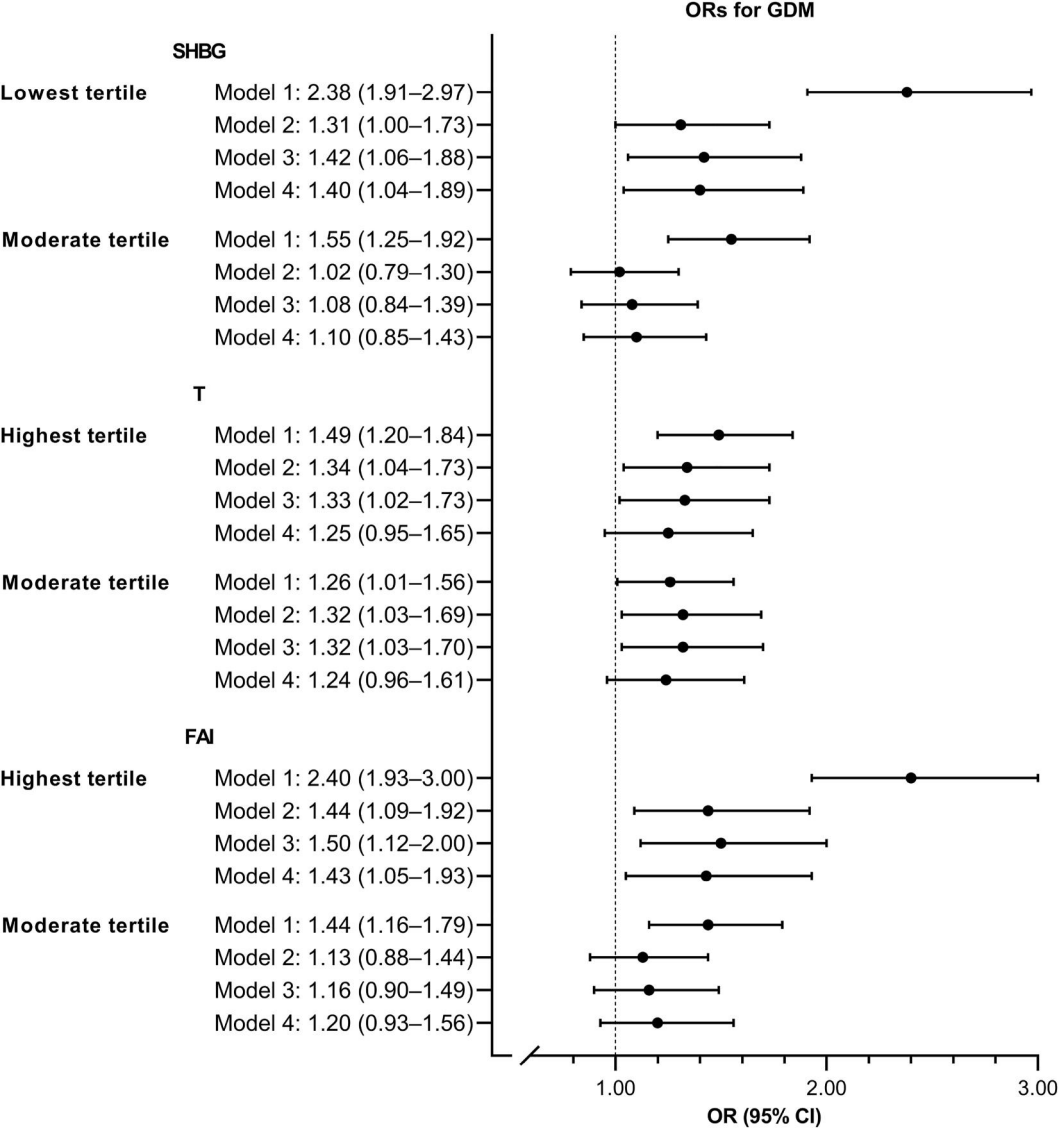
Associations of sex hormone‐binding globulin (SHBG), testosterone (T), and free androgen index (FAI) tertiles with gestational diabetes (GDM) (*n* = 2008). Model 1 was unadjusted. Model 2 was adjusted for gestational weeks at the sampling, pre‐pregnancy BMI, and maternal age. Model 3 was adjusted for the variables in Model 2 and for smoking during pregnancy, educational attainment, parity, delivery hospital, and sex of the newborn. Model 4 was adjusted for the variables in Model 3 and for hypertensive pregnancy complications and gestational weight gain. SHBG: lowest tertile (*n* = 669, range 30.8–202.7 nmol/L), moderate tertile (*n* = 671, range 202.8–284.5 nmol/L), and highest tertile (*n* = 668, range 284.6–703.3 nmol/L). Testosterone: lowest tertile (*n* = 670, range 0.5–2.2 nmol/L), moderate tertile (*n* = 670, range 2.3–3.2), highest tertile (*n* = 668, range 3.3–17.2 nmol/L). FAI: Lowest tertile (*n* = 669, range 0.2–0.9), moderate tertile (*n* = 670, range 1.0–1.4), and highest tertile (*n* = 669, range 1.5–11.3).

Early pregnancy SHBG levels were lower in women who developed GDM than in controls with no GDM (geometric mean 210.5 nmol/L vs. 246.1 nmol/L) (Figure 1). The SHBG level was inversely associated with pre‐pregnancy BMI. After adjustments for gestational weeks at the sampling, pre‐pregnancy BMI and maternal age (Model 2), the SHBG levels were 3.7% (95% CI 0.1%–7.3%) lower in the GDM group than in controls. T was 3.1% (95% CI 0.1%–6.2%) higher and FAI was 4.6% (95% CI 1.9%–7.3%) higher in women with GDM than in controls (geometric means for T 2.8 nmol/L vs. 2.6 and for FAI 1.4 vs. 1.1). After adjustments for covariates in Models 3 and 4, the differences in SHBG and FAI levels remained similar. The higher T levels observed in the GDM group did not significantly differ from those observed in the controls after adjusting for hypertensive pregnancy complications (Model 4). The women in the lowest SHBG tertile and those in the highest FAI tertile had 1.4‐fold odds for GDM compared with those in the corresponding reference tertiles (Figure 2). In additional analyses after excluding eight women with *T* > 10.0 nmol/L or after excluding women with PCOS, the results did not change.

### 3.3 Associations of SHBG, T, and FAI with fasting and post‐prandial hyperglycaemia

**FIGURE 3:**
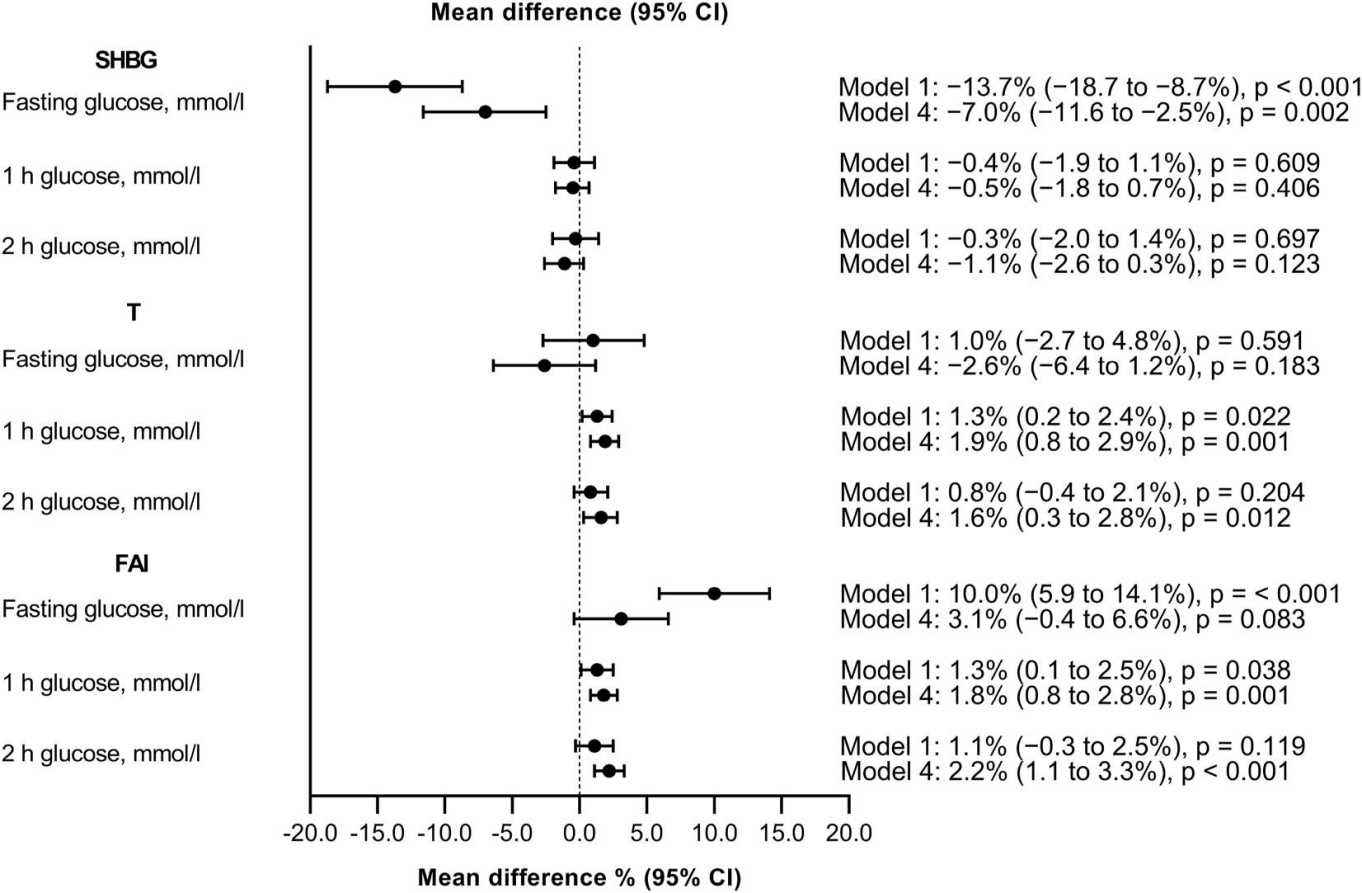
Sex hormone‐binding globulin (SHBG), testosterone (T) and free androgen index (FAI) and their associations with glucose values in a 2 h 75 g oral glucose tolerance test (OGTT) amongst women with gestational diabetes (GDM) (*n* = 1045). Model 1 was unadjusted. Model 4 was adjusted for gestational weeks at the sampling, pre‐pregnancy BMI, maternal age, smoking during pregnancy, educational attainment, parity, delivery hospital, sex of the newborn, hypertensive pregnancy complications, and gestational weight gain.

Amongst the women with GDM, SHBG was inversely associated with fasting glucose (Figure 3). SHBG decreased by 7.0% (95% CI 2.5%–11.6%) when fasting glucose increased by 1 mmol/L, whereas no association between SHBG and 1 or 2 h glucose was observed. Instead, T and FAI were independently associated with higher post‐prandial glucose concentrations during the OGTT. T increased by 1.9% (95% CI 0.8%–2.9%) and FAI by 1.8% (95% CI 0.8%–2.8%) when 1 h glucose increased by 1 mmol/L. Again, T increased by 1.6% (95% CI 0.3%–2.8%) and FAI by 2.2% (95% CI 1.1%–3.3%) when 2 h glucose increased by 1 mmol/L. The results were similar after excluding women with PCOS.

### 3.4 Associations of SHBG, T, and FAI with the severity of GDM

Of the women with GDM, 300 (30.3%) had early‐onset disease (diagnosed <20 weeks of gestation) (detailed characteristics in Table S1), which was independently associated with 6.7% lower SHBG (CI 95% 0.7%–12.7%) and 6.6% higher FAI (CI 95% 2.0%–11.3%) compared with the women diagnosed with GDM later in pregnancy (Table 2).

**TABLE 2:** Differences in sex hormone‐binding globulin (SHBG), testosterone (T) and free androgen index (FAI) levels in women with early‐onset gestational diabetes (GDM) (<20 weeks of gestation) compared with women diagnosed with GDM later in pregnancy

	Early‐onset GDM	
	Yes	No
Number of subjects	300 (30.3%)	690 (69.7%)

Measure and model^a^	Geometric mean (SD)^b^	Geometric mean (SD)^b^	Mean difference (95% CI)	*p* value
SHBG, nmol/L	187.7 (66.4%)	221.9 (55.7%)		
1			−16.7% (−23.0% to −10.4%)	<0.001
2			−6.6% (−12.2% to −0.9%)	0.023
3			−5.3% (−11.1% to 0.4%)	0.067
4			−6.7% (−12.7% to −0.7%)	0.029
T, nmol/L	3.00 (44.9%)	2.75 (38.8%)		
1			6.5% (1.8% to 11.1%)	0.006
2			1.8% (−3.0% to 6.5%)	0.467
3			2.1% (−2.7% to 6.9%)	0.394
4			3.2% (−1.8% to 8.2%)	0.213
FAI	1.68 (52.7%)	1.42 (42.3%)		
1			15.2% (10.2% to 20.3%)	<0.001
2			5.4% (1.0% to 9.9%)	0.017
3			5.1% (0.6% to 9.6%)	0.026
4			6.6% (2.0% to 11.3%)	0.005

^a^ Model 1 was unadjusted. Model 2 was adjusted for gestational weeks at the sampling, pre‐pregnancy BMI, and maternal age. Model 3 was adjusted for the variables in Model 2 and for smoking during pregnancy, educational attainment, parity, delivery hospital, and sex of the newborn. Model 4 was adjusted for the variables in Model 3 and for hypertensive pregnancy complications and gestational weight gain.

^b^ The geometric mean is the *n*th root of the product of *n* values. Geometric SDs correspond to the percent increase in the variable corresponding to a change of one SD unit in the logarithm of the variable.

Of the women with GDM, 197 (19.2%) received anti‐diabetic medication (*n* = 184 insulin, *n* = 22 metformin, *n* = 13 both insulin and metformin) (Table 1). Of the women who needed insulin, 28 (15.2%) received short‐acting, 106 (57.6%) long‐acting, and 47 (25.5%) both short‐ and long‐acting insulin. After adjustments, the early pregnancy SHBG level was 8.7% lower (CI 95% 1.2%–15.4%) and FAI was 7.2% higher (CI 95% 1.7%–12.8%) in the women who needed long‐acting insulin as compared with other women with GDM (Table 3).

**TABLE 3:** Differences in sex hormone‐binding globulin (SHBG), testosterone (T), and free androgen index (FAI) levels amongst women with gestational diabetes (GDM) in those who received any anti‐diabetic medication or long‐acting insulin compared with other women with GDM

Treatment for GDM	Any anti‐diabetic medication				Long‐acting insulin			
	Yes	No			Yes	No		
Number of subjects	197 (19.2%)	827 (80.8%)			153 (14.9%)	871 (85.0%)		

Measure and model^a^	Geometric mean (SD)^b^	Geometric mean (SD)^b^	Mean difference (95% CI)	*p* value	Geometric mean (SD)^b^	Geometric mean (SD)^b^	Mean difference (95% CI)	*p* value
SHBG, nmol/L	197.8 (60.7%)	214.6 (59.1%)			193.4 (62.6%)	214.5 71(58.8%)		
1			−8.1% (−15.4% to −0.9%)	0.028			−10.3% (−18.3% to −2.3%)	0.012
2			−4.9% (−10.9% to 1.1%)	0.113			−8.4% (−15.1% to −1.8%)	0.013
3			−5.1% (−11.1% to 1.0%)	0.099			−8.6% (−15.3% to −2.0%)	0.011
4			−5.4% (−11.8% to 1.1%)	0.102			−8.7% (−15.4% to −1.2%)	0.023
T, nmol/L	2.92 (46.0%)	2.79 (39.5%)			2.89 (41.6%)	2.81 (40.2%)		
1			3.3% (−2.0% to 8.6%)	0.219			2.0% (−3.8% to 7.8%)	0.502
2			3.5% (−1.5% to 8.5%)	0.171			1.5% (−4.1% to 7.1%)	0.597
3			3.8% (−1.3% to 8.8%)	0.145			1.7% (−3.9% to 7.3%)	0.561
4			3.3% (−2.0% to 8.7%)	0.225			2.6% (−3.3% to 8.5%)	0.390
FAI	1.56 (54.0%)	1.37 (43.7%)			1.58 (53.6%)	1.38 (44.3%)		
1			7.9% (2.0% to 13.7%)	0.008			8.3% (1.8% to 14.8%)	0.012
2			6.2% (1.4% to 10.9%)	0.011			6.9% (1.6% to 12.2%)	0.010
3			6.5% (1.7% to 11.2%)	0.008			7.1% (1.8% to 12.4%)	0.008
4			5.9% (0.9% to 10.8%)	0.022			7.2% (1.7% to 12.8%)	0.010

^a^ Model 1 was unadjusted. Model 2 was adjusted for gestational weeks at the sampling, pre‐pregnancy BMI, and maternal age. Model 3 was adjusted for the variables in Model 2 and for smoking during pregnancy, educational attainment, parity, delivery hospital, and sex of the newborn. Model 4 was adjusted for the variables in Model 3 and for hypertensive pregnancy complications and gestational weight gain.

^b^ The geometric mean is the *n*th root of the product of *n* values. Geometric SDs correspond to the percent increase in the variable corresponding to a change of one SD unit in the logarithm of the variable.

## 4 DISCUSSION

This study showed, for the first time, in a large population of pregnant women that SHBG measured in early pregnancy was associated especially with early‐onset GDM, higher fasting glucose, and the need of long‐acting insulin treatment, whereas FAI and T were associated with higher post‐prandial glucose values in the OGTT.

Low SHBG in early pregnancy has been associated with subsequent GDM in previous studies.^17^, ^18^, ^21^, ^23^ The inverse association observed in the present study has been reported to be considerably stronger in some studies,^17^, ^18^, ^21^, ^23^ but most of these previous findings were not adjusted for pre‐pregnancy BMI.^18^, ^21^, ^23^ By contrast, some previous studies did not find this association after taking BMI into account.^22^, ^24^, ^26^ Aside from the lack of adjustments, the controversial results may be related to the small sample sizes (the number of GDM cases varied from 14 to 107) and the considerable heterogeneity of previous studies.^17^, ^18^, ^21^, ^22^, ^23^, ^24^, ^25^, ^26^ We were able to show in this large dataset that low SHBG was also associated with GDM independent of pre‐pregnancy BMI, which is in line with the results of a recent meta‐analysis.^22^

We found that T levels in early pregnancy were slightly higher in women who developed GDM. However, the difference was substantially attenuated when adjusted for pre‐pregnancy BMI, and it was further reduced when adjusted for mediators, such as hypertension in pregnancy. Previous studies disagree on whether elevated T levels in early pregnancy are associated with GDM. Two small studies reported that first trimester T was higher in women who subsequently developed GDM,^17^, ^28^ whereas one study amongst women with PCOS did not find any association.^36^ The early pregnancy T levels reported in this study were comparable to those of a previous study^17^ and published reference values,^37^ but they were higher than those reported in another study.^28^ Of note, the FAI level, a commonly used marker to evaluate hyperandrogenism in women,^38^ was associated with GDM.

In line with previous findings amongst non‐pregnant people,^15^ we found that SHBG was inversely associated with fasting glucose but not with post‐prandial glucose concentrations. It has been suggested that SHBG may affect hepatic gluconeogenesis, which mainly regulates fasting glucose.^14^, ^15^ Conversely, we found that both FAI and T were associated with slightly higher post‐prandial glucose values. In female mice, androgen excess seems to promote chronic androgen receptor activation in pancreatic beta cells, leading initially to increased basal insulin secretion independently of insulin resistance and, eventually, to beta cell failure and hyperglycaemia through oxidative stress.^16^ It has also been reported that hyperandrogenic non‐pregnant women have shown compromised post‐prandial insulin secretion as a possible signal of early beta cell dysfunction,^39^ which may explain our findings.

Women with early‐onset GDM have more clinical risk factors, and they are more insulin resistant already in early pregnancy compared with normoglycemic women with obesity.^8^ This study showed that low SHBG in early pregnancy, which reflects the degree of insulin resistance,^11^ was associated with early‐onset GDM and with the need of long‐acting insulin treatment, which is mostly used for fasting hyperglycaemia. Up to half of the women with GDM develop a glucose metabolism disorder after pregnancy, and especially women who have had fasting hyperglycaemia, early‐onset, or medically treated disease, are at the greatest risk for subsequent type 2 diabetes.^6^, ^10^ It has been suggested that SHBG may play an important role in the pathogenesis of type 2 diabetes by modulating the effects of sex hormones.^40^ Thus, we suggest that the lower levels of SHBG observed amongst women with more severe forms of GDM, especially amongst those with early‐onset and medically treated disease, might represent a more advanced stage in the diabetic cascade.

To the best of our knowledge, this is the largest case–control study evaluating the associations of SHBG, FAI, and T levels in early pregnancy with subsequent GDM and the first study assessing the associations of these parameters with the type of hyperglycaemia estimated using glucose concentrations during an OGTT and severity of GDM, including adjustments for multiple confounders. Moreover, information on the PCOS status of the participants is available. The present results enrich our understanding of the different roles of SHBG and androgen excess with the type and severity of hyperglycaemia during pregnancy as well as provide knowledge of the early steps of the diabetes cascade. Further, the results suggest that SHBG might be helpful for the early recognition of women at high‐risk for early‐onset GDM, which could allow earlier and tailored diagnostic and preventive measures. However, because of the observational study setting, the present results should be interpreted with caution as it is difficult to estimate their effect on clinical outcomes.

There are also limitations to this study. A second‐generation immunoassay was used to determine T concentrations, although liquid chromatography‐tandem mass spectrometry assay is considered the gold standard method for women.^34^ However, the second‐generation immunoassay has been shown to be accurate as the overestimation of the T concentration because of cross‐reaction with other steroids has been related to the first‐generation assays.^34^, ^35^ Based on the reference values of T during pregnancy,^37^ additional analyses were performed after excluding eight women with *T* > 10.0 nmol/L, and the results were similar. Furthermore, early pregnancy serum samples were taken in a non‐fasting state and other steroidal hormones were not measured.

In conclusion, lower SHBG levels in early pregnancy were associated with an increased risk for GDM, especially early‐onset disease, higher fasting glucose, and insulin treatment for fasting hyperglycaemia, whereas high T and FAI levels were associated with higher post‐prandial glucose values. Thus, a low SHBG level may indicate an advanced degree of existing insulin resistance, while androgen excess might reflect impaired post‐prandial insulin secretion. Future clinical follow‐up studies are needed to investigate the roles of these parameters as early signals of the risk of type 2 diabetes after pregnancy.

## AUTHOR CONTRIBUTIONS

Eero Kajantie, Risto Kaaja, Mika Gissler, Johan G. Eriksson, Hannele Laivuori and Marja Vääräsmäki were responsible for the study conception and design of the FinnGeDi study. Eero Kajantie, Laure Morin‐Papunen, Sanna Mustaniemi, Elina Keikkala and Marja Vääräsmäki designed the present study. Hanna Öhman and Heljä‐Marja Surcel were responsible for the laboratory assays. Sanna Mustaniemi performed the data analysis, and all authors contributed to the data interpretation. Sanna Mustaniemi wrote the first draft of the manuscript, and Elina Keikkala, Eero Kajantie, Laure Morin‐Papunen and Marja Vääräsmäki reviewed and edited the manuscript and supervised its writing. All authors critically reviewed the final version of the manuscript and gave their permission for its publication.

## CONFLICT OF INTEREST

The authors have nothing to disclose.

### PEER REVIEW

The peer review history for this article is available at https://publons.com/publon/10.1002/dmrr.3599.

## ETHICS STATEMENT

The study was performed according to the Declaration of Helsinki. The Ethics Committee of Northern Ostrobothnia Hospital District, the Finnish Institute for Health and Welfare and the scientific committee of the Northern Finland Biobank Borealis approved the study.

## Supporting information

Supporting Information S1

Supporting Information S2

## Data Availability

Restrictions apply to the availability of some or all data generated or analysed during this study to preserve patient confidentiality or because they were used under license. The corresponding author will on request detail the restrictions and any conditions under which access to some data may be provided.
