# Supplementary figures and images for: Associations of low sex hormone‐binding globulin and androgen excess in early pregnancy with fasting and post‐prandial hyperglycaemia, gestational diabetes, and its severity
Source: Diabetes Metab Res Rev. 2022 Dec 19;39(2):e3599. doi: 10.1002/dmrr.3599 (PMC10078580; doi:10.1002/dmrr.3599)

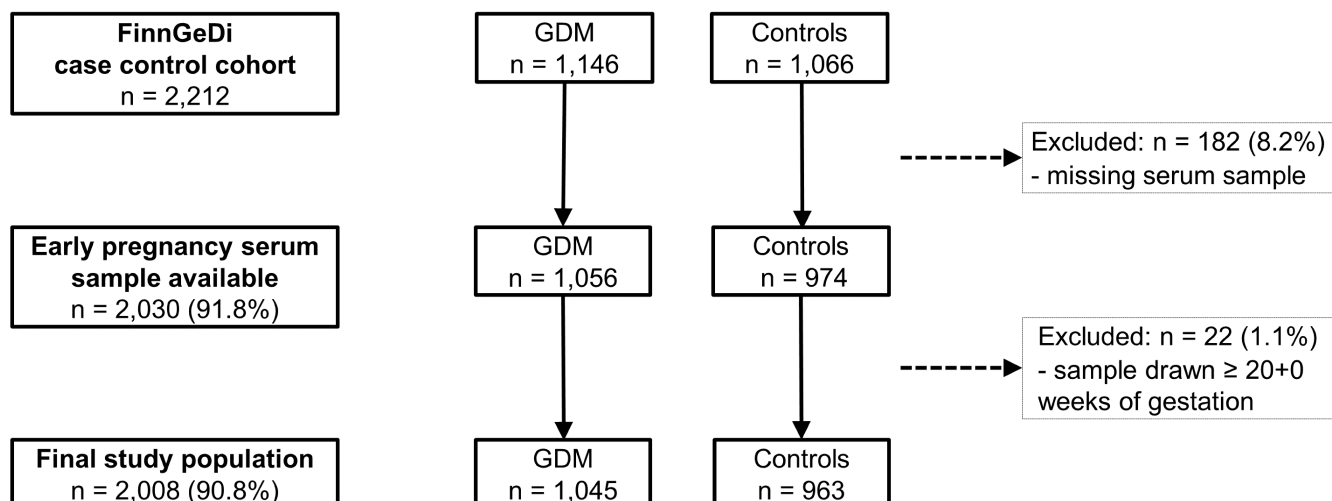

Supplement: Supplementary file 1 — Supporting Information S1 [file DMRR-39-0-s002.pdf]
