# Supplementary material for: Associations of low sex hormone‐binding globulin and androgen excess in early pregnancy with fasting and post‐prandial hyperglycaemia, gestational diabetes, and its severity
Source: Diabetes Metab Res Rev. 2022 Dec 19;39(2):e3599. doi: 10.1002/dmrr.3599 (PMC10078580; doi:10.1002/dmrr.3599)
Supplement: Supplementary file 2 — Supporting Information S2 [file DMRR-39-0-s001.doc]

|  | **Early-onset GDM**  **< 20 weeks of gestation** | | **GDM diagnosed**  **> 20 weeks of gestation** | |  |  |
| --- | --- | --- | --- | --- | --- | --- |
| No. of subjects | 300 (30.3%) | | n = 690 (69.7%) | |  |  |
| **Characteristic** | **Mean (SD)**  **/ n (%)** | **No. of missing** | **Mean (SD)**  **/ n (%)** | **No. of missing** | **P value^a^** | **P value^b^** |
| Age at delivery, years | 33.1 (5.8) | 0 | 31.6 (5.1) | 0 | < 0.001 |  |
| Parity, n | 1.8 (2.4) | 0 | 1.1 (1.7) | 0 | < 0.001 |  |
| Primiparous, n (%) | 78 (26.0%) | 0 | 351 (50.9%) | 0 | < 0.001 |  |
| Pre-pregnancy weight, kg | 86.1 (18.8) | 0 | 72.3 (14.4) | 1 | < 0.001 | < 0.001^c^ |
| Height, cm | 164.8 (6.0) | 0 | 165.0 (5.8) | 0 | 0.651 |  |
| Pre-pregnancy BMI, kg/m^2^ | 31.7 (6.5) | 0 | 26.6 (5.0) | 1 | < 0.001 | < 0.001^c^ |
| Gestational weight gain, kg | 10.1 (6.1) | 22 | 13.3 (5.4) | 54 | < 0.001 | < 0.001^c^ |
| Educational attainment |  | 29 |  | 69 | 0.006 |  |
| Basic or less, n (%) | 20 (6.7%) | 0 | 42 (6.1%) | 0 |  |  |
| Upper secondary, n (%) | 150 (50.0%) | 0 | 267 (38.7%) | 0 |  |  |
| Lower-level tertiary, n (%) | 62 (20.7%) | 0 | 170 (24.6%) | 0 |  |  |
| Upper-level tertiary, n (%) | 39 (13.0%) | 0 | 142 (20.6%) | 0 |  |  |
| Smoking during pregnancy, n (%) | 47 (15.7%) | 0 | 115 (16.7%) | 3 | 0.676 |  |
| PCOS, n (%) | 35 (13.1%) | 32 | 58 (9.5%) | 78 | 0.112 | 0.663^d^ |
| Chronic hypertension, n (%) | 67 (22.3%) | 0 | 88 (12.8%) | 1 | < 0.001 | 0.527^d^ |
| Gestational hypertension, n (%) | 68 (22.7%) | 0 | 139 (20.2%) | 1 | 0.376 | 0.663^d^ |
| Pre-eclampsia, n (%) | 20 (6.7%) | 0 | 37 (5.4%) | 1 | 0.421 | 0.799^d^ |
| Induction of labour, n (%) | 169 (56.3%) | 0 | 273 (39.6%) | 0 | < 0.001 | 0.002^d^ |
| Caesarean section, n (%) | 51 (17.0%) | 0 | 144 (20.9%) | 0 | 0.159 |  |
| Gestational age at delivery, weeks | 39.5 (1.3) | 0 | 39.7 (1.4) | 0 | 0.020 | 0.193^e^ |
| < 37+0 weeks, n (%) | 8 (2.7%) | 0 | 25 (3.6%) | 0 | 0.441 | 0.407^e^ |
| Birth weight, g | 3677 (489) | 0 | 3642 (507) | 0 | 0.319 | 0.613^f^ |
| Birth weight SD score | 0.3 (1.3) | 0 | 0.2 (1.0) | 0 | 0.341 | 0.403^f^ |
| LGA, > +2 SD, n (%) | 22 (7.3%) | 0 | 34 (4.9%) | 0 | 0.132 | 0.845^d^ |
| Use of any anti-diabetic medication (insulin and/or metformin), n (%) | 107 (36.3%) | 5 | 74 (10.9%) | 12 | < 0.001 | < 0.001^d^ |
| Insulin, n (%) | 101 (34.2%) | 5 | 68 (10.0%) | 12 | < 0.001 | < 0.001^d^ |
| Short-acting, n (%) | 12 (4.1%) |  | 12 (1.8%) |  | 0.034 | 0.065^d^ |
| Long-acting, n (%) | 57 (19.3%) |  | 43 (6.3%) |  | < 0.001 | < 0.001^d^ |
| Both, n (%) | 31 (10.5%) |  | 12 (1.8%) |  | < 0.001 | < 0.001^d^ |
| Not specified, n (%) | 1 (0.3%) |  | 1 (0.1%) |  | 0.544 | 0.557^d^ |
| Metformin, n (%) | 13 (4.4%) | 7 | 8 (1.2%) | 15 | 0.001 | 0.016^d^ |

**Table S1.** Maternal and perinatal characteristics of the women with early-onset gestational diabetes (GDM) (< 20 weeks of gestation) compared with women diagnosed with GDM later in pregnancy (n = 990).

^a^ Unadjusted P values based on the Student’s t-test or the χ^2^ test

^b^ Adjusted P values by logistic regression

^c^ Adjusted for parity and maternal age

^d^ Adjusted for parity, maternal age and pre-pregnancy BMI

^e^ Adjusted for parity, maternal age, pre-pregnancy BMI, induction of labour (yes/no) and hypertensive pregnancy complications

^f^ Adjusted for parity, maternal age, gestational age at delivery, pre-pregnancy BMI and hypertensive pregnancy complications

GDM, gestational diabetes; PCOS, polycystic ovary syndrome; LGA, large for gestational age
